# Strategies to Improve the Functional Value of Meat and Meat Products

**DOI:** 10.3390/foods13152433

**Published:** 2024-08-01

**Authors:** Danuta Jaworska, Anna Sadowka

**Affiliations:** Institute of Human Nutrition Sciences, Warsaw University of Life Sciences, Nowoursynowska 159 C Str., 02-776 Warsaw, Poland; anna_sadowska@sggw.edu.pl

Meat is a necessary component of the human diet because of its unique chemical composition, nutritional value, and complete protein content with favourable proportions of amino acids. Meat from slaughter animals is a valuable product in terms of nutritional value, because it provides many nutrients, building and regulating substances. Although meat is commonly perceived as a source of protein, vitamins, and minerals, recent studies indicate that it is also an important source of bioactive substances contributing to the proper functioning of the human body [1].

Meat proteins from slaughter animals are characterised by high bioavailability and high digestibility, ranging from 70 to 95%. They provide all essential amino acids: methionine, lysine, threonine, tryptophan, leucine, isoleucine, phenylalanine, valine, glutamic and aspartic acid, alanine, and arginine. Amino acids are present in proportions that correspond to the needs of the human body. The nutritional and biological value of protein is also influenced by the content of intramuscular connective tissue. Connective tissue proteins do not contain sufficient amounts of exogenous amino acids and are characterised by poorer digestibility. Therefore, the more connective tissue there is in the meat, the lower its quality and the lower its nutritional value. The healthfulness of meat is also influenced by the composition and proportions of fatty acids, which are determined by genetic factors and the animal’s diet. A lower ratio of fatty acids improves immunostimulating functions and prevents coronary heart disease. In beef, the n-6/n-3 ratio is 2.11 (the recommended ratio is from 6:1 to 4:1). Meat contains small amounts of long-chain PUFAs, such as eicosapentaenoic acid, docosapentaenoic acid, and docosahexaenoic acid, which have a potential preventive effect on heart disease. They also reduce the risk of degenerative and metabolic diseases. It is characterised by a higher level of CLA isomers. Fat, in addition to its nutritional properties, influences the development of the culinary characteristics of meat and its organoleptic properties, including palatability. Red meat is rich in trace elements such as iron, selenium, zinc, and copper, which play a key role in many metabolic pathways and enzymatic antioxidant mechanisms [2].

At the same time, a lot of controversy surrounds the high content of saturated fatty acids, so a number of studies are concerned with the development of meat products with reduced fat content, altered fatty acid profile, and reduced cholesterol levels. One of the most important methods of producing such products is to enrich meat products with bioactive components, which include vitamins, minerals, polyunsaturated fatty acids, fibre, phenolic compounds, phytosterols, and oligosaccharides. Other methods relate to changes in the animal’s diet or appropriate handling of the carcass immediately after slaughter. Seven manuscripts (five research, two reviews) related to the above-mentioned subjects have been published in this Special Issue, “Strategies to Improve the Functional Value of Meat and Meat Products”. These manuscripts include an evaluation of meat and carcass quality-related traits in turkey populations through discriminant canonical analysis [3], a fungal biostarter effect on the quality of dry-aged beef [4], and a study on the effect of different heat treatments on the formation of nutritionally unfavourable compounds, assessing the risks and benefits of heat treatment of red meat fixed by different methods [5]. A further four manuscripts deal with the development of the composition and assessment of the quality characteristics of meat products using various functional additives, i.e., burgers with reduced salt content using powders from various types of mushrooms [6], liver sausages enriched with walnut paste [7], and pork patties with a favourable fatty acid composition as a result of the use of developed emulsion gels formulated with a mixture of olive, chia, and algae oils [8]. Interesting results were presented in a review manuscript on the possible use of micro and macro algae and their extracts in meat products [9].

In the work on the study of meat and carcass quality-related traits in turkey populations through discriminant canonical analysis [3], the main differences in meat and carcass quality between turkey genotypes were identified and their clustering patterns were described using discriminant canonical analysis. For this purpose, a comprehensive meta-analysis of 75 publications on turkey carcass and meat traits was conducted. It was concluded that this study could be the starting point for the development of a genotype classification tool based on phenomic traits and could be used as a guide for the evaluation of the literature resources when drawing on the experimental design of research to address the characterisation of meat and carcass quality in a turkey population.

Another study [4] investigated the effect of the fungal biostarter *Mucor flavus* on the physicochemical characteristics and sensory quality of dry-ageing beef. The results showed a significant effect of the fungal biostarter on increasing pH and myosin chain proteolysis and improving sensory quality. *M. flavus* can be used as an inoculum to standardise the dry maturation of beef.

The heating process is a key step that can lead to the formation of harmful chemical compounds in red meat, such as heterocyclic aromatic amines, N-nitrosamines, polycyclic aromatic hydrocarbons, and acrylamide. The aim of the review by Iammarino et. al. (2024) [5] was to present current research on the effects of different heat treatments of red meat on the formation of toxic compounds and nutritional value. The most commonly studied method of heat treatment of meat is grilling. During this process, most of the above-mentioned toxic compounds are formed. Considering the content of adverse toxic compounds, cooking is the best heat treatment of meat, as it causes only a slight increase in polycyclic aromatic hydrocarbons and heterocyclic aromatic amines content. It also causes an unfavourable decrease in PUFAs and vitamins and minerals. Microwave heating and the use of the sous vide method, apart from a slight formation of heterocyclic aromatic amines, do not lead to a significant formation of toxic compounds, and show an additional improvement in the nutritional quality of meat. The nutritional value of heat-treated meat varies between different animal species. This is particularly true for the content of saturated, monounsaturated, and polyunsaturated fatty acids. Heating processes play an important role in improving protein digestibility in red meat from different animal species. Numerous studies confirm that heat treatment of red meat under relatively mild conditions (<100 °C) is most beneficial, preserving nutritional value while avoiding significant formation of toxic compounds.

Another published study evaluated the chemical, physicochemical, and sensory properties of low-sodium beef burgers formulated with the addition of powder from different types of mushrooms as salt replacements [6]. The addition of mushroom powder had no effect on fat and protein content and enabled a 55-61% reduction in sodium content compared to the control sample. Compared to the control sample, the developed burgers had higher shrinkage and weight loss values during cooking; at the same time these samples showed higher lipid oxidation values. In terms of sensory performance, the substitution of sodium chloride with different mushroom powders had no effect on any of the evaluated discriminants. The results obtained in this study indicate that the use of mushroom powder is suitable for use as an alternative to sodium chloride in the production of beef burgers.

In the work of Florowski et. al. (2022) [7], it was hypothesised that walnut paste could be used to design health-promoting liver sausages with favourable quality characteristics with an increased content of bioactive compounds. Walnut paste, as an ingredient with a high fat content, resulted in reduced hardness and increased spreadability of the developed products and also contributed to a favourable change in their colour. On the other hand, a negative aspect of the liver product with the addition of a large amount of walnut paste (i.e., 25%) was the faster oxidation of fats, which would need to be taken into account when developing this type of product on a larger scale.

A manuscript on the effect of replacing pork fat with emulsion gels on the quality characteristics of pork patties [8] describes the development of new emulsion gels containing a mixture of olive oil, chia, and algae emulsified with soy protein isolate and stabilised with two different cold gelling agents, gelatine and chitosan, and evaluates their potential use as a replacement for pork fat in pork patties. Emulsion gels containing a mixture of olive, chia, and algae oils emulsified with soy protein isolate and stabilised with gelatine or chitosan proved to be a suitable partial or full fat replacement in pork patties. Significant reductions in fat content and increases in PUFA and EPA+DHA content as well as reductions in SFA and n-6/n-3 ratios were obtained in the reformulated products compared to the control without negative effects on sensory attributes.

The aim of the literature review carried out in the manuscript “Micro- and Macroalgae in Meat Products” [9] is to systematically describe the effects of including whole algae and their extracts in various meat products, investigating their impact on quality, physicochemical and functional properties, sensory attributes, and the potential for extending shelf life. The addition of algae to meat products generally causes an increase in pH values, with changes depending on concentration, type, initial pH, and storage time. Algae contributed to lower moisture content and higher ash content due to the presence of significant amounts of dietary fibre. Although the addition of algae improved water-holding capacity and reduced cooking losses, it often led to increased hardness and poorer chewability. Algae and their extracts adversely affected the colour of the products, depending on the type of algae. They clearly affected the sensory properties, reducing the overall acceptability of the evaluated products. It is noteworthy that some species of algae had a positive effect on the microbiota of meat products during refrigerated storage, while the use of a 0.1% polysaccharide extract from algae can achieve the same or better effect than commonly used traditional preservatives. It was also found that micro- and macro-algae content of up to 3% could potentially reduce lipid oxidation in meat products. At the same time, algal extracts showed an overall strong antioxidant effect in the developed products.

In summary, it can be concluded that the manuscripts presented in this Special Issue “Strategies to Improve the Functional Value of Meat and Meat Products” contribute important and interesting new knowledge from a cognitive and application point of view and are in line with current nutritional trends for obtaining meat and meat products with high health-promoting properties while maintaining sensory properties and consumer acceptability. Moreover, the consumption of a high-value product such as meat contributes to acceleration of the human body’s metabolism. Today, it is also an important component of the diet because of the nutritional requirements of the body and the presence of many biologically active substances. It should be emphasised that an appropriate supply of bioactive compounds ensures the optimal growth and development of the human body and may also have a preventive effect on many civilization diseases.

However, it should be noted that the negative impact of meat on the human body is often attributed to products produced during heat treatment and improper treatments applied during its processing. Improperly conducted heat treatment (too high temperature—over 180 °C, too long heat process duration, or an inappropriate smoking method) may contribute to the formation of PAHs (polycyclic aromatic hydrocarbons) and HAAs (heterocyclic aromatic amines) with carcinogenic properties [10].
